# Epicardial Adipose Tissue and IL-13 Response to Myocardial Injury Drives Left Ventricular Remodeling After ST Elevation Myocardial Infarction

**DOI:** 10.3389/fphys.2020.575181

**Published:** 2020-10-15

**Authors:** Valentina Parisi, Serena Cabaro, Vittoria D’Esposito, Laura Petraglia, Maddalena Conte, Pasquale Campana, Gerardo Gerundo, Marianna Abitabile, Andrea Tuccillo, Maria Accadia, Giuseppe Comentale, Emanuele Pilato, Mario Sansone, Dario Leosco, Pietro Formisano

**Affiliations:** ^1^Department of Translational Medical Sciences, University of Naples Federico II, Naples, Italy; ^2^Casa di Cura San Michele, Maddaloni, Italy; ^3^URT “Genomics of Diabetes,” Institute of Experimental Endocrinology and Oncology, National Research Council, Naples, Italy; ^4^Department of Emergency Medicine, Ospedale del Mare, Naples, Italy; ^5^Department of Cardiology, Ospedale del Mare, Naples, Italy; ^6^Department of Advanced Biomedical Science, University of Naples Federico II, Naples, Italy; ^7^Department of Electrical Engineering and Information Technology, University of Naples Federico II, Naples, Italy

**Keywords:** left ventricular remodeling, epicardial adipose tissue, inflammation, cytokines, biomarkers, echocardiography

## Abstract

**Introduction:**

Left ventricular (LV) remodeling after ST-segment elevation myocardial infarction (STEMI) is explained only in part by the infarct size, and the inter-patient variability may be ascribed to different inflammatory response to myocardial injury. Epicardial adipose tissue (EAT) is a source of inflammatory mediators which directly modulates the myocardium. EAT increase is associated to several cardiovascular diseases; however, its response to myocardial injury is currently unknown. Among inflammatory mediators, IL-13 seems to play protective role in LV regeneration, but its variations after STEMI have not been described yet. Purpose: In the present study we analyzed the association between infarct-related changes of EAT and IL-13 in post-STEMI LV remodeling.

**Methods:**

We enrolled 100 patients with STEMI undergoing primary angioplasty. At the enrolment (T0) and after 3 months (T1), we measured EAT thickness by echocardiography and circulating levels of IL-13 by ELISA.

**Results:**

At T1, the 60% of patients displayed increased EAT thickness (ΔEAT > 0). ΔEAT was directly associated to LV end-diastolic volume (*r* = 0.42; *p* = 0.014), LV end-systolic volume (*r* = 0.42; *p* = 0.013) and worse LV ejection fraction (LVEF) at T1 (*r* = −0.44; *p* = 0.0094), independently of the infarct size. In the overall population IL-13 levels significantly decreased at T1 (*p* = 0.0002). The ΔIL-13 was directly associated to ΔLVEF (*r* = 0.42; *p* = 0.017) and inversely related to ΔEAT (*r* = −0.51; *p* = 0.022), thus suggesting a protective role for IL-13.

**Conclusion:**

The variability of STEMI-induced “inflammatory response” may be associated to the post-infarct LV remodeling. ΔEAT thickness and ΔIL-13 levels could be novel prognostic markers in STEMI patients.

## Introduction

Left ventricular (LV) remodeling is a complication that occurs after ST-segment elevation myocardial infarction (STEMI) in almost half patients during the first year after the event. Several evidences suggest that it is mainly caused by an inflammatory response to myocardial injury which drives cardiomyocytes apoptosis and myocardial fibrosis (Sutton and Sharpe, 2000; Rodriguez-Palomares et al., 2019) leading to LV enlargement and systolic function worsening. Epicardial adipose tissue (EAT) is the visceral fat depot of the heart; in coronary artery disease (CAD), it has been described as a local source of inflammatory mediators (Mazurek et al., 2003). We previously reported that EAT thickness is increased in CAD patients (Parisi et al., 2020a,c) and the dynamic changes of its secretory activity are important features of acute coronary syndromes, mainly ascribed to the anti-inflammatory mediators (Parisi et al., 2020b). Indeed, despite several pro-inflammatory cytokines have been investigated in the context of LV adverse remodeling, clinical trials failed to show benefits of their inhibition on heart failure patients (Westman et al., 2016). Fewer evidences explore the role of anti-inflammatory cytokines and their modulation. In animal models, IL-13 demonstrated a protective role in cell cycle and heart regeneration via activation of ERK1/2 and Akt signaling, important pathways known to protect against apoptosis and to promote cardiomyocytes proliferation (Wodsedalek et al., 2019). Moreover, interleukin-13 receptor a1 (IL-13Ra1) gene is downregulated in the hearts of patients with end-stage heart failure, suggesting a role for IL-13 in myocardial homeostasis and repair (Amit et al., 2017). Thus, our purpose is to investigate whether myocardial ischemia may be associated to EAT thickness, by echocardiographic evaluation, and to anti-inflammatory response, by measuring circulating IL-13 levels. Hence, we hypothesize that the “inflammatory remodeling” could be associated to the inter-patient variability of LV post-infarct evolution.

## Materials and Methods

### Study Population

The study population included 100 consecutive patients, with STEMI undergoing primary angioplasty, the gold standard therapy as it guarantees immediate restore of blood flow to a blocked artery. The inclusion criteria were: STEMI caused by atherothrombotic CAD (type 1 myocardial infarction as defined by the current guidelines) (Thygesen et al., 2018); primary angioplasty within the times indicated by the current guidelines as treatment (Ibanez et al., 2018); availability of clinical and echocardiographic follow-up at 3 months. The exclusion criteria were: other types of myocardial infarction; previous myocardial revascularization; previous myocarditis; any relevant cardiac or valvular disease; presence of other pathologic conditions associated with morphologic and functional EAT changes, such as aortic valve stenosis (Parisi et al., 2015; Davin et al., 2019) and atrial fibrillation (Wong et al., 2017); hemodynamic instability; presence of cancer and or systemic inflammatory diseases which might affect EAT and/or circulating inflammatory profile.

Baseline demographic, clinical, and echocardiographic data, including EAT thickness, were collected at the admission in emergency department (T0) and after 3 months (T1). We also collected a blood sample for determination of IL-13 circulating levels at T0 and T1.

In order to explore the relationship between circulating and EAT levels of IL-13, and to better define EAT as a source of IL-13 in CAD, we enrolled in the study 55 patients undergoing coronary artery by-pass grafting and we collected blood samples and EAT biopsies for IL-13 determination (D’Esposito et al., 2020) (Supplementary Material).

The study was approved by the local Ethics Committee. All procedures performed in the study were in accordance with the ethical standards of the institutional or national research committee and with the 1964 Helsinki declaration and its later amendments or comparable ethical standards and conformed to the Declaration of Helsinki on human research. All patients included in the study gave written informed consent after receiving an accurate explanation of the study protocol and of the potential risks related to the procedures adopted by the study.

### Echocardiographic Study

Echocardiograms were performed by a VIVID E9 (GE Healthcare) machine, according to standard techniques. All patients underwent a complete echocardiographic examination including EAT measurement at T0 and T1. We evaluated LV systolic and diastolic function, left atrial dimensions, and excluded any relevant valvular disease. LV volumes and ejection fraction were considered as parameters of remodeling at T1.

Epicardial adipose tissue thickness was obtained, as previously described and validated (Parisi et al., 2020c). EAT was visualized in parasternal long-axis view between the free wall of the right ventricle and the anterior surface of the ascending aorta. To improve image quality, the depth was reduced, the focus adjusted, and ultrasound beam frequency slightly increased. The colorimetric map was switched into gold. Once visualized the EAT deposit, the maximum EAT thickness was measured at end-systole. The average value from three cardiac cycles was used for the statistical analysis. The ΔEAT was obtained by subtracting EAT at T0 from EAT at T1.

### IL-13 Determination

We collected blood samples for serum IL-13 determination at T0 and T1. IL-13 circulating levels were determined by using the Bioplex Multiplex human cytokine assay (Bio-Rad, Hercules, CA, United States) according to the manufacturer’s instructions. IL-13 levels in EAT, mature adipocytes and stromal vascular fraction cells secretomes were determined as described in Supplementary Material.

### Statistical Analysis

Numerical variables have been summarized using median and percentiles [25th; 75th] or as media ± standard deviation while absolute frequencies and percentages have been used for categorical factors.

Comparisons between paired groups were based on the Wilcoxon signed-rank test. Correlations among variables were assessed using the Pearson correlation coefficient. A *p* value <0.05 was considered significant for all tests. Partial correlation coefficient has been used for assessing the correlation between two variables independently from a third variable. All analyses were performed using R (R Core Team, 2019).

## Results

### Study Population

N.100 consecutive patients with STEMI were enrolled in this study. A total of 19 patients were lost at follow-up; 11 patients had previous acute coronary syndromes or revascularizations and four patients had concomitant systemic inflammatory diseases. Thus, 66 individuals were included in the final analysis. Clinical and demographic characteristics of the study population are reported in Table 1.

**TABLE 1:** Demographic and clinical data of the study population.

	Study population
Age (years)	62.5(54;69)
Gender, male n (%)	55 (83)
BMI	26.12(24.69;27.1)
Creatinine (mg/dl)	0.89(0.73;1.0)
Glucose (mg/dl)	133(117;163)
Cholesterol (mg/dl)	180(156;213)
Myoglobin (ng/ml)	183(67;324)
Creatine kinase MB (U/l)	64(19;113)
Troponin I (ng/ml)	0.6(0.11;3.8)
GFR (ml/min)	92(72;103)
Hypertension, *n* (%)	37 (56.1)
Diabetes, *n* (%)	12 (18.2)
Smokers, *n* (%)	53 (80.3)
Dyslipidemia, *n* (%)	15 (22.7)
Beta blockers, *n* (%)	4 (6.1)
Calcium antagonists, *n* (%)	7 (10.6)
ACE inhibitors, *n* (%)	20 (30.3)
Sartan, *n* (%)	1 (1.5)
Statin, *n* (%)	4 (6.1)
Antiaggregants, *n* (%)	6 (9.1)
PTCA LAD, *n* (%)	32 (48.5)
PTCA CX, *n* (%)	6 (9.1)
PTCA RCA, *n* (%)	29 (43.9)

*BMI, body mass index; GFR, glomerular filtration rate; ACE, angiotensin-converting enzyme; PTCA, percutaneous transluminal coronary angioplasty; LAD, left anterior descending artery; CX, circumflex artery; RCA, right coronary artery.*

The mean age was 62.5 years, 56% of patients were hypertensives, 18% were diabetics, 80% were smokers, and 22% were dyslipidemics. At the discharge all patients received the standard medical therapy and behavioral suggestions recommended by the current guidelines (Ibanez et al., 2018).

Echocardiography was performed at baseline (T0) and 3 months after STEMI (T1). Echocardiographic parameters are reported in Table 2. At T0, left ventricular end-diastolic volume (LVEDV) was 84 ml [71; 101]; left ventricular end-systolic volume (LVESV) was 40 ml [33; 50]; left ventricular ejection fraction (LVEF) was 53% [45; 56]. At T1, LVEDV was 96 ml [75; 112]; LVESV was 39 ml [30; 54]; LVEF was 55% [49; 60].

**TABLE 2:** Echocardiographic data of the study population.

	Study population
LVEDV, ml	93 (74.5; 108)
LVESV, ml	39 (30; 51)
LVEF, %	55 (49.5; 61.5)
LVEDD, mm	48 (45; 52.3)
LVDSD, mm	35 (30; 39)
Septum, mm	9.5 (9; 11)
Posterior wall, mm	9 (8; 10)
Left atrial volume, ml/m^2^	46 (40; 55)
E/A	0.9 (0.7; 1.3)
E/e′	9.4 (7.6; 12.9)
TAPSE, mm	24.5 (22; 27.3)
SPAP, mmHg	27.5 (25; 30)
MR grade 0; 1; 2; ≥3 *n* (%)	10 (15.2); 42(63.6); 14 (21.2); 0 (0)
AR grade 0; 1; 2; ≥3 *n* (%)	27 (40.9); 37 (56.1); 2 (3); 0 (0)

*LVEDV, left ventricular end-diastolic volume; LVESV, left ventricular end-systolic; LVEF, left ventricular ejection fraction; LVEDD, left ventricular end-diastolic diameter; LVESD, left ventricular end-systolic diameter; TAPSE, tricuspid annular plane systolic excursion; SPAP, systolic pulmonary artery pressure; MR, mitral regurgitation; AR, aortic regurgitation.*

### Epicardial Adipose Tissue and Left Ventricular Remodeling

**FIGURE 1:**
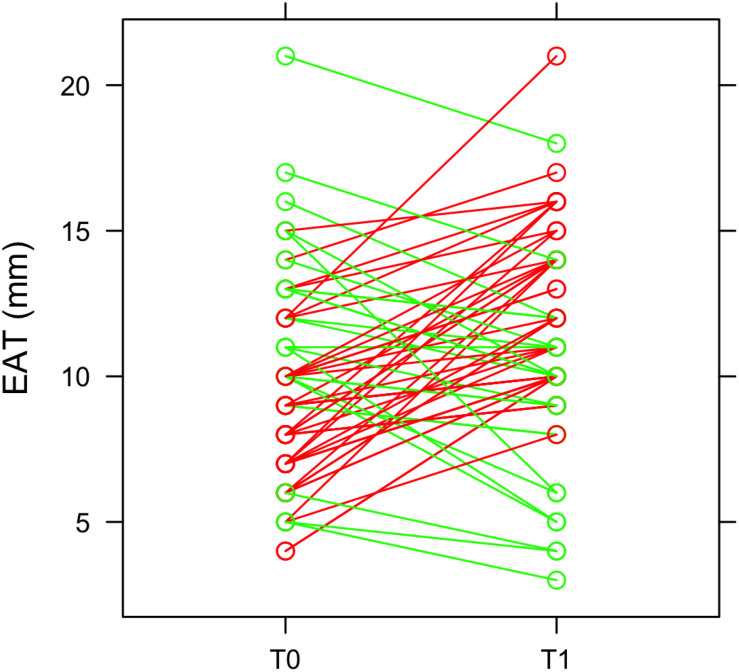
EAT thickness and heterogeneous remodeling. EAT thickness was obtained by echocardiographic examination at the admission in emergency department (T0) and after 3 months (T1). The green lines represent patients in which EAT thickness decreases at T1, while the red lines refer to patients with an increase of EAT thickness at T1.

At 3 months after STEMI (T1) we clearly observed a heterogeneous EAT remodeling in the study population (EAT thickness at T0: 9.6 ± 3.3; EAT at T1: 11.2 ± 3.4; *p* = 0.011). In some patients EAT thickness increased and, in some others, decreased (Figure 1). In particular, 43 patients (65%) had an EAT increase at 3 months. EAT changes were not paralleled by a significant change in body mass index (BMI) (T0 = 26.12 kg/m^2^; T1 = 25.88 kg/m^2^; *p* = 0.072). Among patients with a ΔEAT <0 and ΔEAT > 0 no differences in demographic and clinical parameters were evidenced Supplementary Material).

**FIGURE 2:**
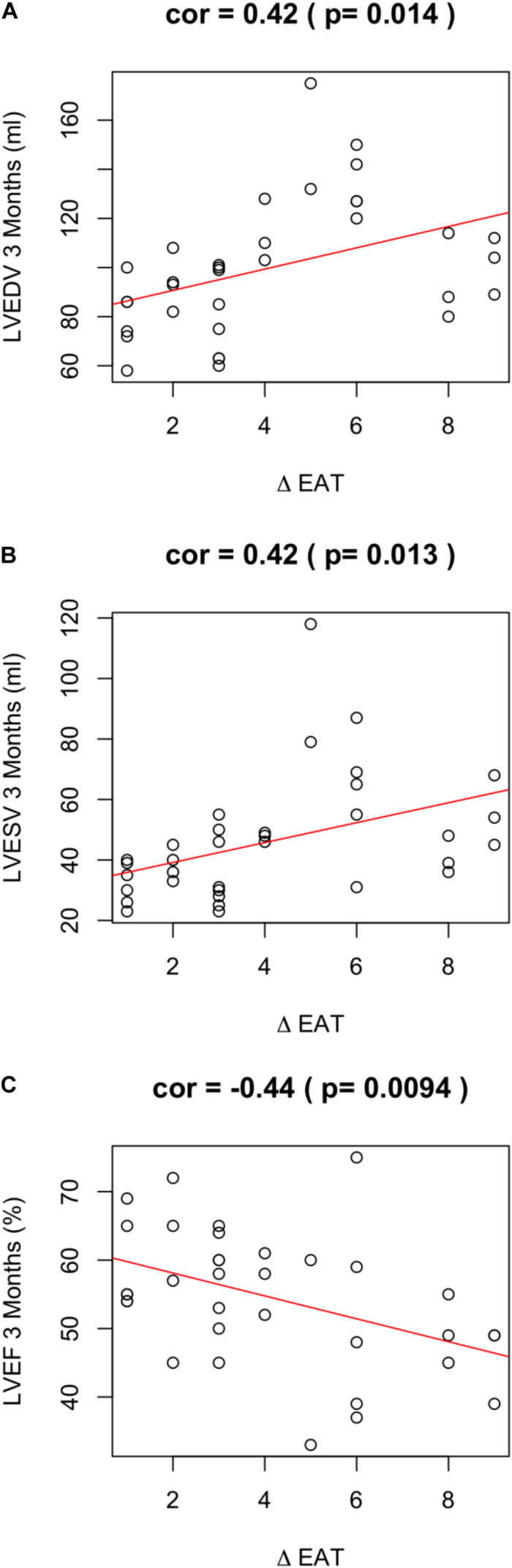
ΔEAT and LV remodeling. Relationship between ΔEAT and **(A)** LVEDV (*r* = 0.42; *p* = 0.014), **(B)** LVESD (*r* = 0.42; *p* = 0.013), and **(C)** LVEF (*r* = −0.44; *p* = 0.0094) was assessed using the Pearson correlation coefficient.

Of note, EAT enlargement was associated to worse LV remodeling. In particular, a positive ΔEAT was directly associated to increased LVEDV (*r* = 0.42; *p* = 0.014) (Figure 2A), increased LV end systolic volume (LVESV) (*r* = 0.42; *p* = 0.013) (Figure 2B). Thus, the increase of EAT thickness after myocardial infarction was associated to LV dilation at 3 months. Of note, this observation was confirmed when we assessed the partial correlation coefficients between delta-EAT and LVEDV at 3 months after STEMI, independently from the site of percutaneous transluminal coronary angioplasty (PTCA). For this purpose, we tested the two more frequent PTCA sites in our population: left anterior descending artery (LAD) and right coronary artery (RCA) (*r* = 0.35, *p* = 0.0003 and *r* = 0.35, *p* = 0.004, respectively). Thus, we can hypothesize that the possible influence of ΔEAT on LV remodeling is independent from the infarct localization.

A positive ΔEAT was also associated to worse LVEF at 3 months (*r* = −0.44; *p* = 0.0094) (Figure 2C). LVEF is expression of LV systolic function and is the major predictor of outcome in infarcted patients. Of note, ΔEAT predicted LVEF at T1 independently of maximal troponin levels during the admission (*r* = −0.466; *p* = 0.04). This finding suggests that the association between EAT increase and impaired LVEF is independent from the infarct size and strengthen the role of inflammation in LV remodeling. Interestingly, however, nor EAT at T0 neither EAT at T1 were predictors *per se* of LV remodeling or systolic function at 3 months after STEMI (EAT-T0 and LVEDV-T1 *r* = −0.22, *p* = 0.082; EAT-T0 and LVESV-T1 *r* = −0.18, *p* = 0.162; EAT-T0 and LVEF-T1 *r* = 0.11, *p* = 0.365; EAT-T1 and LVEDV-T1 *r* = 0.14, *p* = 0.281; EAT-T1 and LVESV-T1 *r* = 0.08, *p* = 0.531; EAT-T1 and LVEF-T1 *r* = −0.14, *p* = 0.267).

### IL-13 and Left Ventricular Remodeling

**FIGURE 3:**
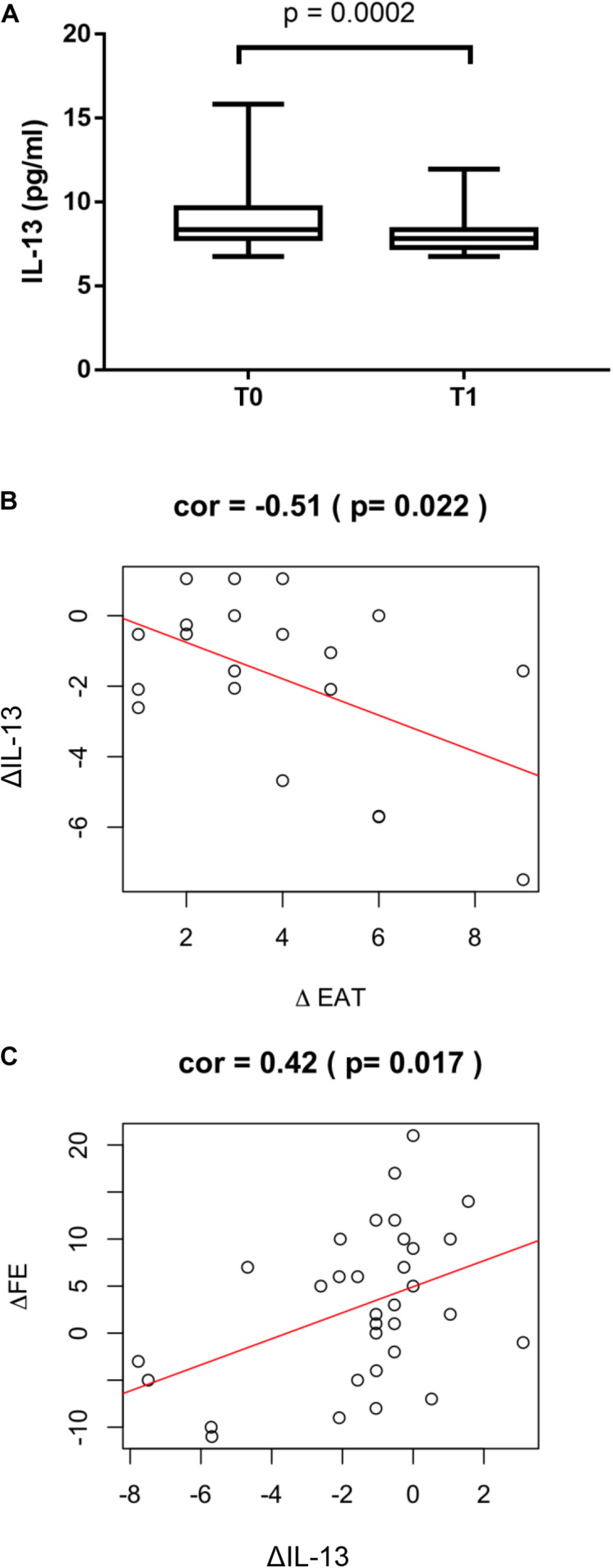
ΔIL-13 and LV remodeling. **(A)** IL-13 serum levels were measured by using the Bioplex multiplex assay kit as described in section “Materials and Methods”. Values are expressed in pg/ml and their distribution within each group is represented by box plot. Relationship between ΔIL-13 and **(B)** ΔEAT (*r* = −0.51; *p* = 0.022) and **(C)** ΔLVEF (*r* = 0.42; *p* = 0.017) was assessed using the Pearson correlation coefficient.

Circulating levels of IL-13 were measured to test the possible favorable role of this anti-inflammatory cytokine in post STEMI LV remodeling. In the overall study population IL-13 levels significantly decreased at T1 compared to T0 (*p* = 0.0002, Figure 3A). The reduction at T1 of IL-13 was associated to a positive ΔEAT, consistent with increased local inflammation (*r* = −0.51; *p* = 0.022) (Figure 3B). Of interest, ΔIL-13 was directly associated to ΔLVEF (*r* = 0.42; *p* = 0.017) thus suggesting a favorable role for IL-13 in myocardial remodeling post STEMI (Figure 3C). In the surgical group a significant correlation between circulating and EAT levels of IL-13 was found (*r* = 0.51, *p* < 0.001) thus suggesting that the peripheral levels may mirror EAT inflammatory status (Supplementary Material).

## Discussion

The main findings of the present study are: 1) There is an inter-patient variability of EAT remodeling after acute myocardial infarction; 2) EAT increase seems to be associated to post-infarct LV dilation and reduced systolic function; 3) EAT increase seems to be associated to reduced levels of IL-13; 4) IL-13 seems to favorably affect myocardial recovery as its increased levels are associated to higher LVEF.

Although most STEMI patients undergo primary angioplasty within the recommended time frame, a subgroup develop progressive adverse LV remodeling and heart failure (Westman et al., 2016). The variability in post-infarct LV dilatation cannot be attributed exclusively to the infarct size and, in patients who survive the first days, a large infarct is neither necessary nor sufficient for progressive adverse LV remodeling (Westman et al., 2016). Inflammation is a critical component of tissue healing. Different modulation of the inflammatory response may explain LV remodeling in certain subgroups of patients.

Epicardial adipose tissue shares a close anatomic proximity and vascular supply with the heart (Iacobellis, 2015) and directly modulate the myocardium through the secretion of inflammatory mediators (Mazurek et al., 2003). Its accumulation has been associated with the onset and progression of CAD (Mahabadi et al., 2013; Nakanishi et al., 2014). Of note, echocardiographic EAT thickness predicts major adverse cardiovascular events in CAD patients (Morales-Portano et al., 2018). EAT can be measured with different imaging methods and, among all, cardiac magnetic resonance (CMR) is considered the gold standard from many authors. However, echocardiography is certainly the more simple and diffuse methodology, available in the acute settings. We previously validated the echocardiographic method for EAT thickness determination used in the present study against CMR and we reported an excellent intra and inter observer variability in CAD patients (Parisi et al., 2020c).

In this prospective study conducted on STEMI patients we evaluated changes in EAT thickness, measured at the Rindfleisch fold (Parisi et al., 2020c), at baseline and at 3 months after the event. The presence of a standardized protocol for the management of STEMI and the strictly inclusion and exclusion criteria allowed us to study in a homogeneous population both myocardial and inflammatory remodeling after the injury. Interestingly, we observed a very variable response of EAT at 3 months from myocardial infarction with a clear identification of a group of patients with EAT thickening. No significant changes of BMI were detected at T1. The finding of a non-parallel trend between EAT and BMI is not surprising. Visceral and subcutaneous adipose tissues differ in accumulation and metabolism and the relationship between EAT and anthropometric parameters is still debated. Our and other groups previously reported that EAT increase is independent from BMI in patients with cardiovascular diseases (Parisi et al., 2015; Villasante Fricke and Iacobellis, 2019).

The significant change in EAT accumulation early after a myocardial injury suggest a bidirectional cross-talk between EAT and the myocardium, and different possible EAT responses to similar stimuli (myocardial ischemia). Many studies reported that EAT thickness, being a marker of EAT inflammatory status, is associated to increased cardiovascular risk, but this is the first evidence of EAT remodeling following myocardial injury. In the present population a ΔEAT > 0 was associated with higher LV volumes and lower LV systolic function, independently from the troponin peak, which has been used to estimate the infarct size. Cardiac troponins provide very useful information in this respect, especially in patients with STEMI (Hallén, 2012). Troponin peak has been demonstrated to be an independent predictor of infarct size measured at SPECT in a community-based cohort of patients with first myocardial infarction (Arruda-Olson et al., 2011). Thus, we can hypothesize that the myocardial ischemia drives EAT phenotypic changes which in turn regulate post-infarct LV remodeling. This hypothesis is also supported by the evidence that neither basal nor at follow-up EAT thickness were associated by themselves to LV post-infarct remodeling. EAT may transduce systemic inflammation to the myocardium and have a crucial role in the balance between inflammatory and anti-inflammatory cytokines. IL-13 is an anti-inflammatory cytokine that promotes polarization of macrophages toward an M2 phenotype (Gause et al., 2013; Wynn, 2015). IL-13 directly acts on target tissues to promote cell cycle activity during regenerative wound healing (Kasaian and Miller, 2008). Previous evidence demonstrated a role for endogenous IL-13 in neonatal cardiomyocytes cell cycle and heart regeneration and suggested recombinant IL-13 as therapeutic approach for activating pro-regenerative and survival pathways in the heart (Wodsedalek et al., 2019). Furthermore, IL-13 could be particularly involved in post-ischemic myocardial remodeling by suppressing fibrinogen production and inhibiting dendritic cell and/or macrophage function (Vasse et al., 1996; Davidson et al., 2007; Shao et al., 2007). Jafarzadeh et al. (2009) reported higher circulating levels of IL-13 in control subject compared to infarcted patients. In the current study, our data are consistent with a protective role of IL-13, as its increase after myocardial infarction is associated to recovery of LV systolic function at 3 months. We also observed that changes in IL-13 circulating levels at 3 months are inversely associated to EAT thickening. In the surgical group (Supplementary Material) we also observed that EAT and its cell subtypes are a source of IL-13. We also observed a significant correlation between EAT and serum levels of IL-13. This evidence supports the hypothesis that serum levels mirror EAT inflammatory status. However, we cannot exclude that the relationship between local and systemic inflammation could be bidirectional and circulating levels of inflammatory mediators could concur to EAT phenotypic and functional remodeling. The possible link between IL-13 and EAT remodeling has been explored in the context of valvular heart disease, were EAT thickness is associated to adverse LV remodeling (Vianello et al., 2019). In patients with valvular heart disease, IL-13 expression by EAT is involved in molecular up-regulation of genes encoding for fat mediators associated with adipose tissue remodeling (Vianello et al., 2019). The finding that only the ΔEAT thickness, and not EAT at T0 or T1, is associated to LV adverse remodeling in our study population, can be explained by the acute nature of the pathology considered. While in valvular heart disease, the progression of the disease goes parallel with the increase of EAT thickness, STEMI is an acute event affecting both myocardial and EAT remodeling.

In conclusion, the evaluation of post-STEMI “inflammatory remodeling” could help in the risk stratification for STEMI patients. In this regard, as echocardiography is a routine examination in CAD, EAT thickness should be routinely measured in order to appreciate its early increase after an acute event.

## Study Limitations

The small sample size and the observational nature of the present study prevented us to achieve definitive conclusions and causative mechanisms. In order to investigate the possible underlying physio-pathological mechanisms and strengthen the hypothesis of a role of EAT in myocardial remodeling, we analyzed EAT biopsies from CAD patients undergoing surgery. We recognize that the findings obtained in this population cannot prove any causality of the observed result and further studies are required to clarify the mechanisms by which EAT influence LV remodeling after STEMI. Further studies are also required to investigate the time-course of IL-13 and other cytokines in order to confirm and better clarify the role of inflammatory mediators on LV remodeling in the acute settings.

Epicardial adipose tissue thickness measurement can be obtained by different methodologies. Certainly, echocardiography is most suitable in the acute setting. However, many different echocardiographic methods have been described and it is not known whether they are interchangeable. The method used in the present work has been previously validated in CAD patients against CMR and offers an excellent reproducibility. It remains unknown whether the same results could be replicated using different echocardiographic methods.

## Data Availability Statement

The raw data supporting the conclusions of this article will be made available by the authors, without undue reservation.

## Ethics Statement

The studies involving human participants were reviewed and approved by University of Naples Federico II. The patients/participants provided their written informed consent to participate in this study.

## Author Contributions

VP and SC were the main contributors in terms of conception, design, acquisition and interpretation of data, and in drafting the manuscript. LP, MC, PC, and GG organized the database. VD’E and SC performed the cytokine assay. VP, MAb, AT, MAc, GC, and EP enrolled the patients and performed echocardiographic analysis. MS performed the statistical analysis. PF and DL mainly contributed in terms of conceptual design, interpretation and discussion of the results, and supervision of the overall work. All authors contributed to the manuscript revision, read, and approved the submitted version.

## Conflict of Interest

The authors declare that the research was conducted in the absence of any commercial or financial relationships that could be construed as a potential conflict of interest.
