# Supplementary material for: Epicardial Adipose Tissue and IL-13 Response to Myocardial Injury Drives Left Ventricular Remodeling After ST Elevation Myocardial Infarction
Source: Front Physiol. 2020 Oct 15;11:575181. doi: 10.3389/fphys.2020.575181 (PMC7593695; doi:10.3389/fphys.2020.575181)
Supplement: Supplementary file 1 [file Table_1.docx]

**Supplementary materials.**

***Surgical group***

In order to explore the relationship between circulating and EAT levels of IL-13, and to better define EAT as a source of IL-13 in CAD, we enrolled in the study 55 patients undergoing coronary artery by-pass grafting. The median age was 66 (61; 73); 78 % were male; median BMI was 26.7 (24.8; 30.1) kg/m^2^; 85% were hypertensives; 64% were dyslipidemics; 53 % were diabetics and 45 % were smokers.

Before cardiac surgery, we collected blood samples for serum IL-13 determination. Intraoperative EAT biopsies (average 0.1–0.5 g) were collected before the initiation of cardiopulmonary bypass, just after the opening of the pericardial sac, between the free wall of the right ventricle and the anterior surface of the ascending aorta.

The study was approved by the local Ethics Committee. All procedures performed in the study were in accordance with the ethical standards of the institutional or national research committee and with the 1964 Helsinki declaration and its later amendments or comparable ethical standards and conformed to the Declaration of Helsinki on human research. All patients included in the study gave written informed consent after receiving an accurate explanation of the study protocol and of the potential risks related to the procedures adopted by the study.

***Circulating IL-13 determination.***

IL-13 circulating levels were determined by using the Bioplex Multiplex human cytokine assay (Bio-Rad, Hercules, CA, United States) according to the manufacturer’s instructions.

***EAT IL-13 determination.***

Mature adipocytes and stromal vascular fraction (SVF) were obtained in 5 patients as previously described by our group (32). Secretomes were obtained from whole tissue and from cells as follows: tissues were weighted, cut into small pieces, and transferred into a 12-well plate. According to tissue weight, serum-free DMEM (1 mL medium/0.1 g tissue) was added to the well and incubated at 37 °C in a CO2 incubator. Mature adipocytes and SVF were incubated in serum-free DMEM. After 24 h, medium was collected and centrifuged at 14,000g to remove debris and analyzed for IL-13 content by using the Bioplex Multiplex human cytokine assay (Bio-Rad, Hercules, CA, United States) according to the manufacturer’s instructions.

***Epicardial adipose tissue as a direct source of IL-13.***

EAT was confirmed to be a source of IL-13. IL-13 median value in EAT secretome was 2.73 (2.35; 3.38) pg/ml. The median value of circulating IL-13 in this population was 11,46 (9.38; 15.57) pg/ml. Of interest we found a significant correlation between local and systemic levels of IL-13 in this population (r=0.51, p<0.001). Thus, we can hypothesize that IL-13 circulating levels may mirror EAT inflammatory status.

***Cells source of EAT IL-13.***

In order to better understand the cell source of EAT derived IL-13 we isolated mature adipocytes and SVF from EAT biopsies and measured IL-13 levels in their secretomes. We observed that IL-13 is produced by both cell types, with a relevant contribution of mature adipocytes (median value 3 pg/ml).

**Table 3**: Demographic and clinical data of the study population

|  | ΔEAT ≤ 0 (24) | ΔEAT > 0 (43) | p value |
| --- | --- | --- | --- |
| Age (years) | 65.5 (52.5; 70) | 62 (54; 69) | 0.699 |
| Gender, male n (%) | 17 (70.1) | 4 (90.1) | 0.053 |
| BMI | 26.12 (24.72; 27.47) | 26.12 (24.69; 27.24) | 0.873 |
| Creatinine (mg/dl) | 0.92 (0.72; 1.03) | 0.88 (0.76; 1.02) | 0.919 |
| Glucose (mg/dl) | 132 (120.3; 160.8) | 135 (114; 166) | 0.951 |
| Cholesterol (mg/dl) | 174 (147.5; 205) | 186 (164; 219) | 0.441 |
| Myoglobin (ng/ml) | 200.8 (73.8; 367.1) | 175.3 (62.6; 325.5) | 0.704 |
| Creatine kinase MB (U/L) | 64,88 (23.2; 93) | 60 (18; 190.6) | 0.583 |
| Troponin I (ng/ml) | 1.69 (0.08; 4.52) | 0.5 ((0.11; 3.01) | 0.411 |
| GFR (ml/min) | 93.4 (68.5; 102.8) | 92.7 (73.1; 106.7) | 0.763 |
| Hypertension, n (%) | 12 (50) | 26 (60.4) | 0.407 |
| Diabetes, n (%) | 4 (16.6) | 9 (20.1) | 0.672 |
| Smokers, n (%) | 17 (70.8) | 37 (80) | 0.131 |
| Dyslipidemia, n (%) | 4 (16.6) | 11 (25.6) | 0.401 |

*BMI, body mass index; GFR, glomerular filtration rate*
